# EriB targeted inhibition of microglia activity attenuates MPP^+^ induced DA neuron injury through the NF-κB signaling pathway

**DOI:** 10.1186/s13041-018-0418-z

**Published:** 2018-12-18

**Authors:** Fangfang Dou, Xinkun Chu, Bei Zhang, Liang Liang, Guoqiang Lu, Jianqing Ding, Shengdi Chen

**Affiliations:** Department of Neurology and Institute of Neurology, Ruijin Hospital affiliated to Shanghai Jiao Tong University School of Medicine, Shanghai, 200025 China

**Keywords:** Parkinson’s disease, Eriocalyxin B, Microglia-associated inflammation, DA neurons, NF-κB signaling pathways

## Abstract

Accumulating evidence indicates that microglia activation is associated with an increased risk for developing Parkinson’s disease (PD). With the progressive and selective degeneration of dopaminergic (DA) neurons, proinflammatory cytokines are elevated in the substantia nigra (SN) of PD patients. Thus, anti-inflammation has become one of the therapeutic strategies of PD. Eriocalyxin B (EriB), a diterpenoid isolated from *Isodoneriocalyx*, was previously reported to have anti-inflammatory effects. MPTP mouse model and MPP^+^ cell model were prepared to detect the role of EriB in regulating microglia activation and neuron protection. Midbrain tissue and primary cultured microglia and neuron were used to examine microglia activation and neuron damage by immunofluorescence, real-time PCR, western-blot and Elisa assay. Open field activity test was to evaluate the changes of behavioral activity in MPTP-induced PD mouse model. EriB was efficacious in protecting DA neurons by inhibiting microglia activation in PD mice model. Treatment with EriB led to amelioration of disordered sports of PD mice model, which correlated with reduced microglia-associated inflammation and damaged DA neurons. EriB treatment abolished MPP^+^ induced microglia activation damages to DA neurons in a microglia and DA neurons co-culture system. The underlying mechanism of EriB-induced protective effects involved inhibition of microglia associated proinflammatory cytokines production through the phenotypic shift of microglial cells as well as activator of transcription and nuclear factor-κB (NF-κB) signaling pathways. These findings demonstrate that EriB exerts potent anti-inflammatory effects through selective modulation of microglia activation by targeting NF-κB signaling pathways, thus exerting the protective effect against on MPP^+^-induced DA neurons injury. This study may provide insights into the promising therapeutic role of EriB for PD.

## Introduction

Parkinson’s disease (PD) is a common neurodegenerative disorder which is characterized by the loss of dopamine (DA) neurons in the ventral midbrain with cell bodies in the substantia nigra pars compacta (SNpc) [1]. In sporadic PD models, microglial activation related neuroinflammation due to exposure to 1-methyl − 4-phenyl-1,2,3,6-tetrahydropyridine (MPTP) has been implicated in nigral DA neurons loss and the degenerative process [2]. Tumor necrosis factor-α (TNF-α), interleukin-1β (IL-1β), IL-6 and transforming growth factor (TGF) levels were elevated in the substantia nigra DA neurons and also in cerebrospinal fluid (CSF) in sporadic PD [3], and TNF-α and IL-6 were also present in microglia of PD brain [4]. Most evidence confirms that neurodegenerative diseases such as PD and Alzheimer’s disease are associated with neuritis, but the exact effect of inflammation on neuronal survival and whether it is to change PD progression is unclear.

Evidence suggests that over activation of microglia in SNpc is a key factor in DA neuronal death and the occurrence of PD [5, 6]. In certain environmental toxins such as MPTP, paraquat and rotenone stimulation, microglia exhibit excessive activation and release of neurotoxic proinflammatory cytokines and reactive oxygen species (ROS). In astrocytes and microglia, MPTP can be transformed into MPP+ [1- methyl-4-phenyl-pyridinium] by monoamine oxidase B, and then MPP+ causes a series of oxidative stress that induces neuronal death because it was taken up by DA transporters in neurons and enters the mitochondria [7]. Accordingly, microglia plays an important role in the PD model induced by MPTP, however, the direct damage of activated microglia to DA neurons except for the role of MPP+ remains to be studied. Inflammation has been considered as a double-edged sword in the central nervous system (CNS), in acute phase of brain injury, activated microglia protect damaged neurons and try to promote its recovery, however, when the inflammatory reaction enter the chronic phase of the disease, the main function of microglia is to eliminate irreparable neurons [8, 9]. Strong evidence implicates TNF plays a key role in the pathophysiology of PD, not only in early stage but also in the late stage, and it could lead to the release of inflammatory cytokines. Some reports supported the hypothesis that specific pro-inflammatory cytokines IL-6, IL-1β, interferon (IFN), and TNF-α secreted by glioblastoma cells could affect intracellular patterns of alpha-synuclein, tau and ubiquitin, possibly promoting DA neurons loss and the PD regression procedures [10]. Eriocalyxin B (EriB) is a diterpenoid compound extracted from Isodoneriocalyx and is a perennial herb of the Labiatae family in southwestern China. For a long time, it has been widely used in Chinese medicine as anti-inflammatory remediation agent [11]. Previous reports indicate that EriB can induce leukemia and lymphoma cell apoptosis by increasing intracellular ROS levels and inhibiting NF-κB pathway, previous studies have also confirmed that EriB can block TNF-induced NF-κB activation by inhibiting IκB degradation in acute myeloid leukemia cell line Kasumi-1 cells. EriB also changes the intracellular redox state by increasing ROS, which may further modulate the redox-sensitive signaling pathway and transcription factors including NF-κB [12, 13]. The role of EriB in regulating NF-κB and ROS pathways is also described in lymphomas and many other tumor cells [14]. In this study, we discuss the key experimental evidence implicating microglia associated inflammation response in the progressive degeneration of the DA neurons and their potential contribution to the pathological process of PD, and we describe studies performed in the PD mice model that indicate potential therapeutic effects of EriB on PD and delineate underlying molecular mechanisms.

## Material and methods

### MPTP mouse model

**Fig. 1:**
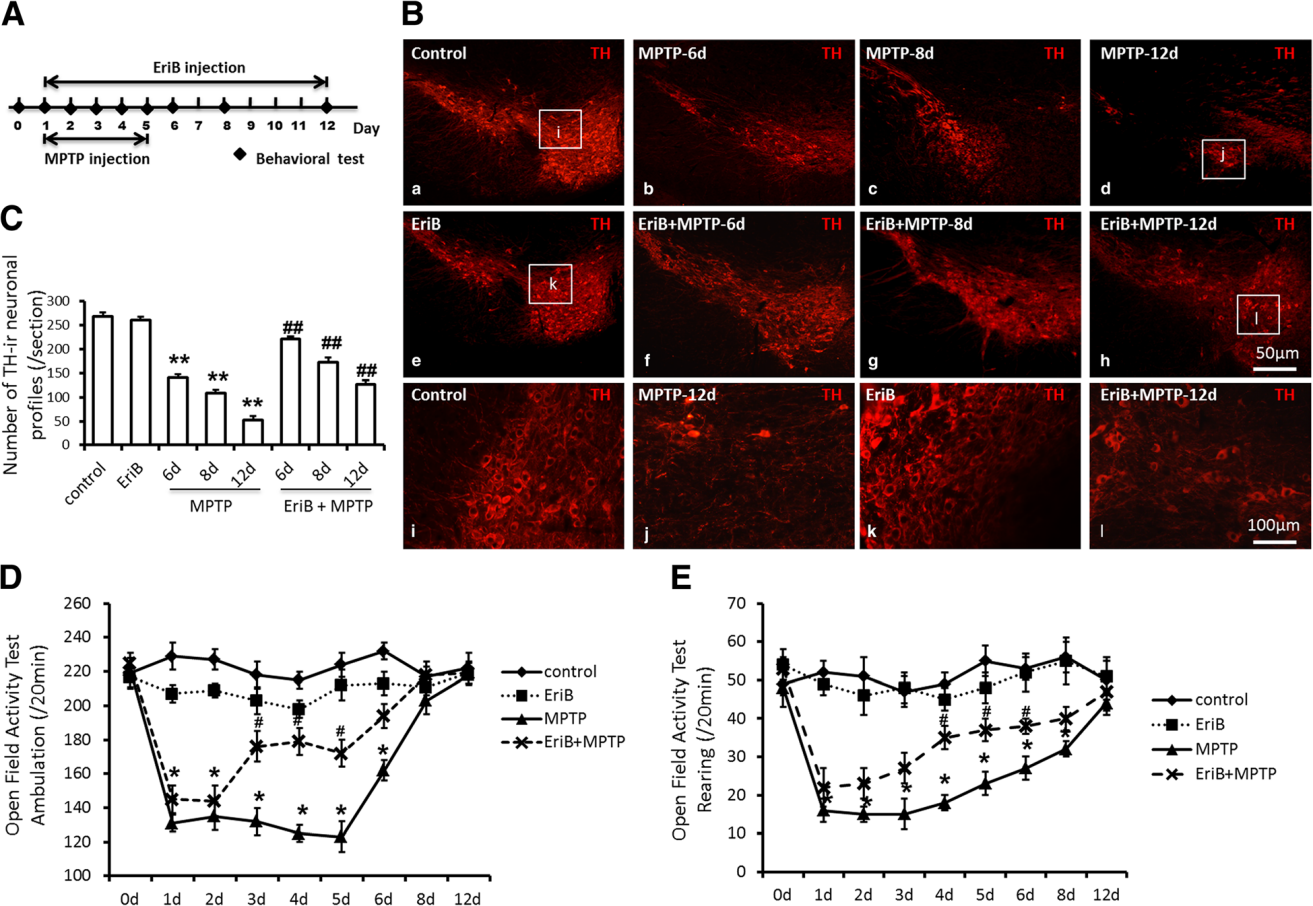
EriB attenuated DA neurons damage and alleviated motor deficits in PD mice model. **a** The time point of MPTP and EriB injection and sample test. **b** Immunohistochemical staining for TH positive DA neurons in SN area after MPTP and EriB injection on different time point. **c** Number of TH positive neurons in SN area after MPTP and EriB injection on different time point. **d-e** Open field activity test of PD mouse after MPTP and EriB injection on different time point. Scale bar: 50 μm, 100 μm. *: vs control, #: vs MPTP group. *&#: *P*<0.05, **&##: *P*<0.01

In order to prepare sub-acute MPTP mouse model, eighty male C57BL/6 mice (8-week-old) were received one injection of 30 mg/kg MPTP (Sigma-Aldrich, MO, USA) daily for five consecutive days intraperitoneally, this regimen causes loss of nigral DA neurons by 40–50% in young adult mice, and it can stabilizes the DA neuron lesion to 21-day after MPTP administration [15]. Totally 104 mice were randomly divided into control group (saline-treated) (13 mice), MPTP-treated group (39 mice), EriB-treated group (13 mice), and MPTP+EriB-treated group (39 mice), and two mice suddenly died after injection of MPTP. Of the 13 mice in each group, 5 were used for pathological sections, 4 for protein extraction, and 4 for RNA extraction. EriB was purchased from KINDU Biotechnology Co., Ltd. (China), EriB (10 mg/kg/day) was injected intraperitoneally daily for twelve days starting on the day when MPTP administration (Fig. 1a) [16].Groups of mice were killed and the severity of DA neurons damage and the activation of microglia were examined at 6 days (13 mice), 8 days (13 mice), and 12 days (13 mice) following MPTP and EriB treatment. MPTP-induced motor deficit was assessed with open field assay at 0, 1, 2, 3, 4, 5, 6, 8, and 12 days following MPTP treatment points. The body shape, hair and mental condition of the mice were observed when we collected the tissues, mice in good condition will be used for subsequent experiments.

### Open field activity test

The open field test is a classical method to evaluate the changes of behavioral activity in MPTP-induced PD mouse model [17, 18]. In our study, a set of infrared beams sent through the open field was used to record mice’s activity. The frequency of beam breaks were took down by software to obtain parameters such as locomotor time, traveled distance, and number of rearings. Open field testing was performed at 2 h after MPTP and/or EriB treatment.

### Immunofluorescence

Immunofluorescence was performed on serial cryostat sections (10 μm, all the SN area) of brain of C57BL/6 mice perfused by 4% paraformaldehyde. Primary rabbit tyrosine hydroxilase (TH) polyclonal antibody (Millipore, Germany, 1:200), primary rabbit Iba-1 polyclonal antibody (Wako, Denmark, Japan, 1:100) (overnight at 4 °C) and primary rabbit p-P65 polyclonal antibody (CST, USA, 1:100) were revealed with specific goat anti-rabbit Alexa Fluor® 488 (IgG H&L) and/or Alexa Fluor® 546 (IgG H&L) (Abcam, USA, 1:200) conjugated secondary antibodies (2 h at room temperature). Cell morphology of DA neurons labeled by TH antibody and microglia labeled by Iba-1 polyclonal antibody was observed under routine fluorescence microscope (CX43 Biological Microscope, Olympus, Japan), microglia morphology labeled by p-P65 polyclonal antibody was observed under laser scanning confocal microscope (LSM 710 System, ZEISS, Germany). Nuclei were labeled with DAPI.

### The number and average fluorescence area of immune positive cells measurement

Primary TH antibody was used to calculate the number TH positive DA neurons in SN area [19]. Ten brain slides of each mouse were selected, and the number of TH-positive neurons was counted and averaged. Briefly, we selected 10 sections from about 60 brain tissue sections in each group for TH fluorescence staining. Photographing the SN and VTA (Ventral tegmental area) regions using a fluorescence microscope (× 100) after dyeing is completed, manually count the number of cells stained for all TH positives in the picture, and then we divided the total number by 10 to average, finally, we will use this value for comparison between groups. Primary Iba-1 antibody and the second-fluorescence antibody were firstly used to detect the quantity and the distribution of microglia in mouse SN area in vivo and primary cultured microglia in vitro. Then, the microglia fluorescence pictures were collected by fluorescence microscope. The principle of collecting pictures: (1) 5 mice in each group, (2) 10 tissue sections of each mouse, (3) 1 tissue section picked from 5 serial tissue sections, (4) cultured primary microglia staining pictures obtained from 3 different experiments at least, (5) 10 fields were selected from each of the fluorescence stained sections. Image Pro Plus 6.0 software (Mediacybernetics, MD, USA) was used to analyze the fluorescence area of Iba-1 immune positive cells.

### Real-time PCR assay

Total RNA (1 μg) extracted from midbrain of C57BL/6 mice was reverse-transcribed into cDNA using a PrimeScript™ II 1st Strand cDNA Synthesis Kit (TAKARA, Shiga Prefecture, Japan) according to the manufacturer’s instructions. Real-time PCR was performed using SYBR® Premix Ex Taq™ (TliRNaseH Plus) (TAKARA, Shiga Prefecture, Japan). Reactions were amplified and analyzed by mean of an ABI 7900 Real-time PCR System (Thermo Fisher Scientific, Waltham, USA).A comparative threshold cycle (Δ*C*_T_) method was used to quantify target mRNAs at different time points. GAPDH was used as internal reference gene. PCR primers were as follows: IFN-γ (forward: 5’-AGGAACTGGCAAAAGGATGG-3′; reverse: 5’-CAGGTGTGATTCAATGACGC-3′), TNF-α (forward: 5’-GGTTCATGTTAACCAGGCCA-3′; reverse: 5′- CCCTCCAGAAAAGACACCATG-3′), IL-1β (forward: 5’-ACGGACCCCAAAAGATGAAG-3′; reverse: 5’-TTCTCCACAGCCACAATGAG-3′), IL-6 (forward: 5’-CAAAGCCAGAGTCCTTCAGAG-3′; reverse: 5’-GTCCTTAGCCACTCCTTCTG-3′), MHC-II(forward: 5’-GCCCAGAGAATTCAGACCAG-3′; reverse: 5’-ACATTATAGCGCAGAGTGTG-3′), MCP-1(forward: 5’-GTCCCTGTCATGCTTCTGG-3′; reverse: 5’-GCTCTCCAGCCTACTCATTG-3′), MIP-1α (forward: 5’-GGTATTCATCTTTGGTTTTGTGGG-3′; reverse: 5’-AATGGTAGTGTGAGCAGGAAG-3′), IP-10 (forward: 5’-TCAGCACCATGAACCCAAG-3′; reverse: 5’-CTATGGCCCTCATTCTCACTG-3′), iNOS (forward: 5’-GCAAACATCACATTCAGATCCC-3′; reverse: 5’-TCAGCCTCATGGTAAACG-3′), CD86 (forward: 5’-GTAGAGTCCAGTTGTTCCTGTC-3′; reverse: 5’-TGGTTCTGTACGAGCACTATTT-3′), Arg1 (forward: 5’-GTCCCTAATGACAGCTCCTTTC-3′; reverse: 5’-CCACACTGACTCTTCCATTCTT-3′), CD206 (forward: 5’-GTGGTCCTCCTGATTGTGATAG-3′; reverse: 5’-CACTTGTTCCTGGACTCAGATTA-3′), GAPDH (forward: 5’-CTTTGTCAAGCTCATTTCCTGG-3′; reverse: 5’-TCTTGCTCAGTGTCCTTGC-3′).

### Western-blot assay

Western-blot analysis was performed as previous described [20]. Different primary polyclonal anti-iNOS, anti-Arg1, anti-IκBα (inhibitor of NF-κB), anti-phosphor-IκBα, anti-p65, anti-phosphor-p65, anti-p38, anti- phosphor-p38 and anti-tubulin antibody were all purchased from cell signaling technology (CST, USA) and used in the present study. After a secondary horseradish peroxidase (HRP)-conjugated goat anti-rabbit/mouse immunoglobulin (Ig) G antibody (1:10,000; SABC) incubated for 1 h at 37 °C, signals were detected by the enhanced chemiluminescence (ECL) kit (Pierce, Rockford, IL, USA).

### Primary DA neuron and microglia culture

C57BL/6 mice ventral mesencephala neurons at gestation day 14 were dissociated and cultured in vitro using a modified method [21]. In brief, mesencephala were placed in cold PBS (phosphate buffer saline) (-Ca^+ 2^/Mg^+ 2^) and cut into small pieces, The tissue was transferred to a 15 ml tube and incubated in 0.1% Trypsin-EDTA (Invitrogen, Germany) and 0.02% DNase I (Roche, Germany) at 37 °C for 15 min in a shaking incubator water bath. The trypsin-EDTA was removed and replaced with 10 ml Dulbecco’s modified Eagle’s medium (DMEM, Sigma, Germany). The digested cells were lightly and repeatedly pipetted using a 5 mL pipette, filtered through 100 mesh filter, and single cells mixed with DMEM supplied with 10% fetal bovine serum (FBS), glucose (30 mM), glutamine (2 mM), 1% penicillin-streptomycin, Hepes buffer and amphotericin were plated onto coverslips coated with 200 μg/mL poly-L-lysine and incubated at 37 °C, 5% CO2. From the 3th day in vitro (DIV), neuron culture medium was replaced by serum-free Neurobasal [96% Neurobasal-A (Life Technologies, Califonia, USA), 1% N-2 supplement, 2% B27 and 1% penicillin–streptomycin]. At DIV 10, either MPP+ (25 μM) (Sigma Aldrich, USA) or EriB (0.25 μM) was added to primary neuron cultures.

Microglia was prepared from C57BL/6 mice postnatal 3–5 days [22]. In brief, cerebral cortex tissues were collected into HBSS-Hank’s solution (Thermo Fisher, USA) in sterile containers. After the meninges were totally removed from cerebral cortex, tissues were chopped and transferred into 0.125% trypsin-EDTA (Thermo Fisher, USA) for 15 min at 37 °C, then trypsinization was stopped with 10% FBS (Thermo Fisher, USA). After mechanical triturating and further centrifugation, the cells were resuspended in the Dulbecco’s modified Eagle medium /F12 (DMEM/F12) (Thermo Fisher, USA) with 10% FBS and plated them onto 175 cm^2^ flasks. Every 5 mice were used per 175 cm^2^ flask. After 2 weeks, the flasks of mixed neuron-glia cultures were shaken at 250 rpm for 4 h at 37 °C to achieve microglia which loosely growth on the astrocytes. The medium was collected to obtain purified microglia which was re-suspended in DMEM/F12 medium and placed in 24-wellplates containing 100 μg/ml poly-L-lysine. At DIV 3, either MPP+ (25 μM) or EriB (0.25 μM) was added to primary microglia cultures.

### Cell viability assay

Cell viability was evaluated with the CCK-8 kit (Beyotime Biotechnology, China) in 96-well plates. Primary DA neuron and microglia were respectively incubated with 20 μl CCK-8 for 2 h at 37 °C according to the manufacturer instructions. Cell viability was analyzed using a microplatereader to determine the absorbance at 450 nm (Biotec, Germany). The obtained values were presented as folds of the controls.

### Enzyme linked immunosorbent assay

Supernatants from cultured primary microglia were collected at 24 h to measure concentrations of IFN-γ, TNF-α, IL-1β and IL-6 using cytokine kit detected by microplate reader according to the manufacturer’s instructions. All of the cytokines were purchased from R&D system (Minneapolis, MN, USA).

### Statistical analysis

Statistical analysis was performed using SPSS 18.0 software (SPSS Inc., Chicago, USA). Data are presented as mean ± standard deviation (SD) of three independent experiments. One-way ANOVA with the appropriate Tukey’s post hoc t-tests was used to compare experimental group, and a *P*-value less than 0.05 was considered statistically significant.

## Results

### EriB attenuated DA neurons damage and alleviated motor deficits in PD mice model

To determine whether EriB play a role in neuron protection in MPTP mouse model, we examined the status of TH positive neurons damage in SN area of mouse brain. Histological analysis of brain tissue sections from normal control mice showed intact SN tissue structure and healthy DA neurons (Fig. 1b, a & i), whereas severe DA neurons damage and loss were observed at the 6th, 8th, and 12th days in MPTP-treated mice (Fig. 1b, b, c, d & j). Therefore, we would like to observe whether EriB has a protective effect on DA neurons in MPTP PD mouse firstly. For the treatment regimen, EriB and MPTP administration starting simultaneously in adult mice to determine the protective role of EriB by measuring the numbers of injured DA neurons and the mean behavioral activity score compared with normal control. The results of immunohistochemistry showed that EriB administration remarkably attenuated DA neurons damage in PD mice (Fig. 1b, f, g, h & l), in addition, EriB administration alone did not lead to DA neurons injury (Fig. 1b, e & k). Figure 1c showed the number of TH positive neurons in each group, on the 6th day (140.6c neurons), 8th day (106.8 ± 8.7 neurons) and 12th day (53 ± 9.8 neurons) after MPTP injection, DA neurons were reduced by 47, 58, and 76% compared with normal control (269.5 ± 9.6 neurons), respectively, however, the DA neurons in the SN region were respectively increased by 29, 25 and 28% in EriB treatment group at the 6th (221.8 ± 10.6 neurons), 8th (173.2 ± 11.2 neurons), and 12th days (127.9 ± 9.1 neurons) in PD model. EriB administration alone does not damage DA neurons (261.4 ± 6.2 neurons). These results demonstrated the protective effect of EriB on MPTP-induced DA neurons injury.

Based on the protective effect of EriB on MPTP-induced DA neurons injury, we expected to further evaluate the quality effect of EriB on PD mice model by means of behavioral score. In the majority of research, hypokinesia was defined as a decrease of locomotion and/or rearing function of mice following treatment with MPTP. We detected the locomotion function of C57BL/6 mice using open field assay to observe the influence of the deficits of motor function with dopamine neurons loss and to assess the role of EriB in attenuating the motor deficit severity of PD model. With the losses of DA neurons, the numbers of ambulation and rearing were both decreased with MPTP (131.8 ± 9.7 and 16.3 ± 7.1 on 1th day) administration, however, EriB increased the locomotion and rearing function from the 3th day (176.4 ± 19.2) to 5th day (172.3 ± 17.4) and 4th day (35.9 ± 8.6) to 6th day (38.5 ± 6.2) respectively following EriB and MPTP administration, after 6 days or 8 days of treatment, we found no significant differences in locomotion or rearing among the four groups (Fig. 1d & e). Our results indicated that EriB could improve motor deficits of MPTP-induced PD mouse model.

### EriB reduced microglia activation by inhibiting proinflammatory cytokines production and shifting phenotypic of microglia

**Fig. 2:**
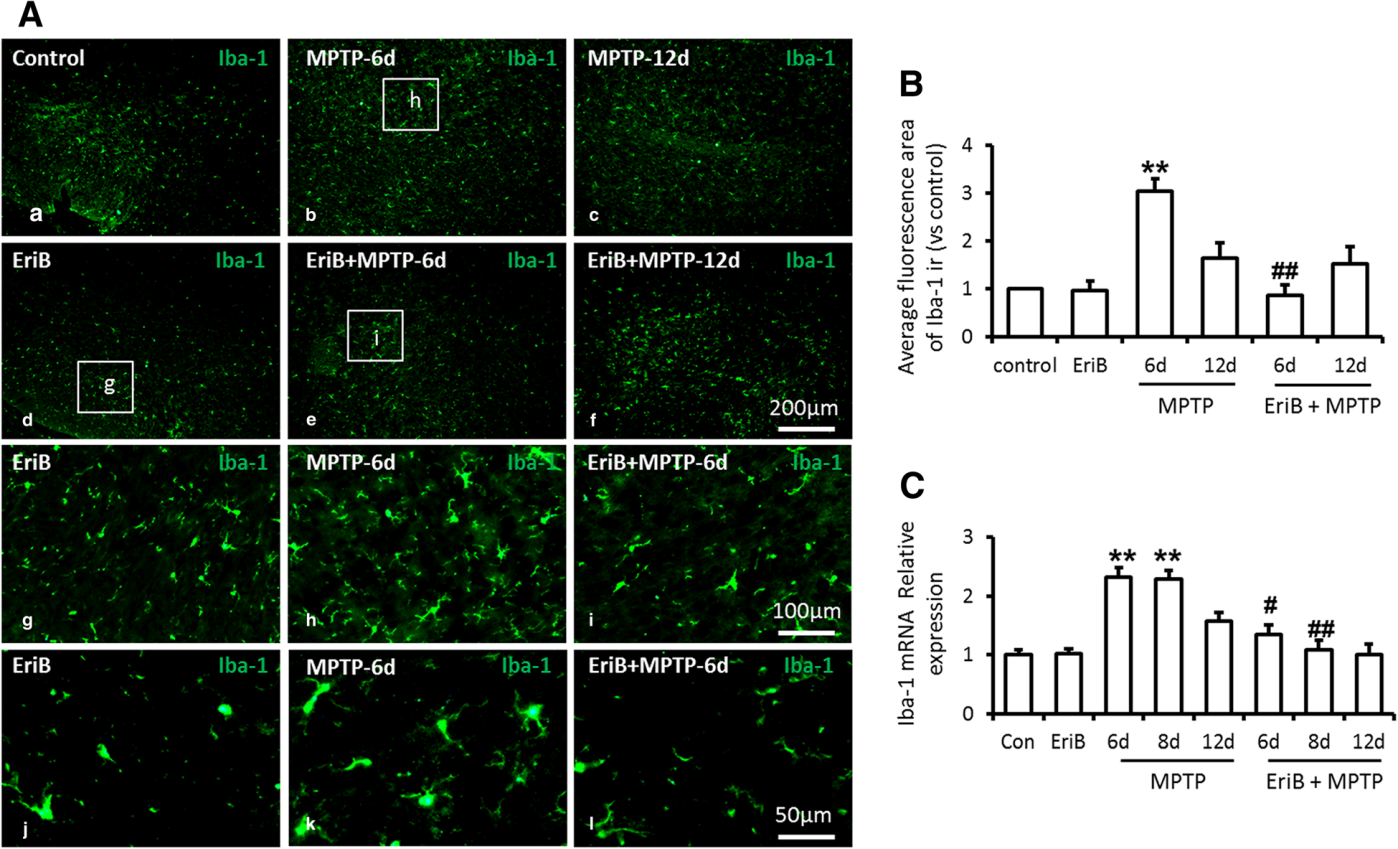
EriB inhibited microglia activation in PD mice model. **a** Immunohistochemical staining for Iba-1 positive microglia in SN area after MPTP and EriB injection on different time point. **b** Average Iba-1 immune-fluorescence area in SN area after MPTP and EriB injection on different time point. **c** mRNA level of Iba-1 after MPTP and EriB injection on different time point. Scale bar: 50 μm, 200 μm. *: vs control, #: vs MPTP group. *&#: *P*<0.05, **&##: *P*<0.01

How did EriB protect against MPTP-induced nigral DA neurons injury and improve the motor deficit in PD mouse model? Based on the anti-inflammatory effects of EriB, we firstly examined the activated status of microglia in SN area of mice brain sections. Iba-1 staining showed that the number and the volume of microglia increased significantly with MPTP treatment (Fig. 2a, b, c & h), however, EriB significantly inhibited MPTP induced activation of microglia by decreasing the number and the volume of activated microglia (Fig. 2a, e, f & i) compared with the normal control. EriB administration alone did not lead to microglia activation (Fig. 2a, d & g). Through the statistics of average of fluorescence area of Iba-1 staining, we confirmed EriB could inhibit the activation of microglia at 6 days rather than 12 days in MPTP mouse model (Fig. 2b), and the mRNA level of Iba-1 was also decreased by EriB treatment on the 6th day and 8th day in MPTP mice (Fig. 2c).

**Fig. 3:**
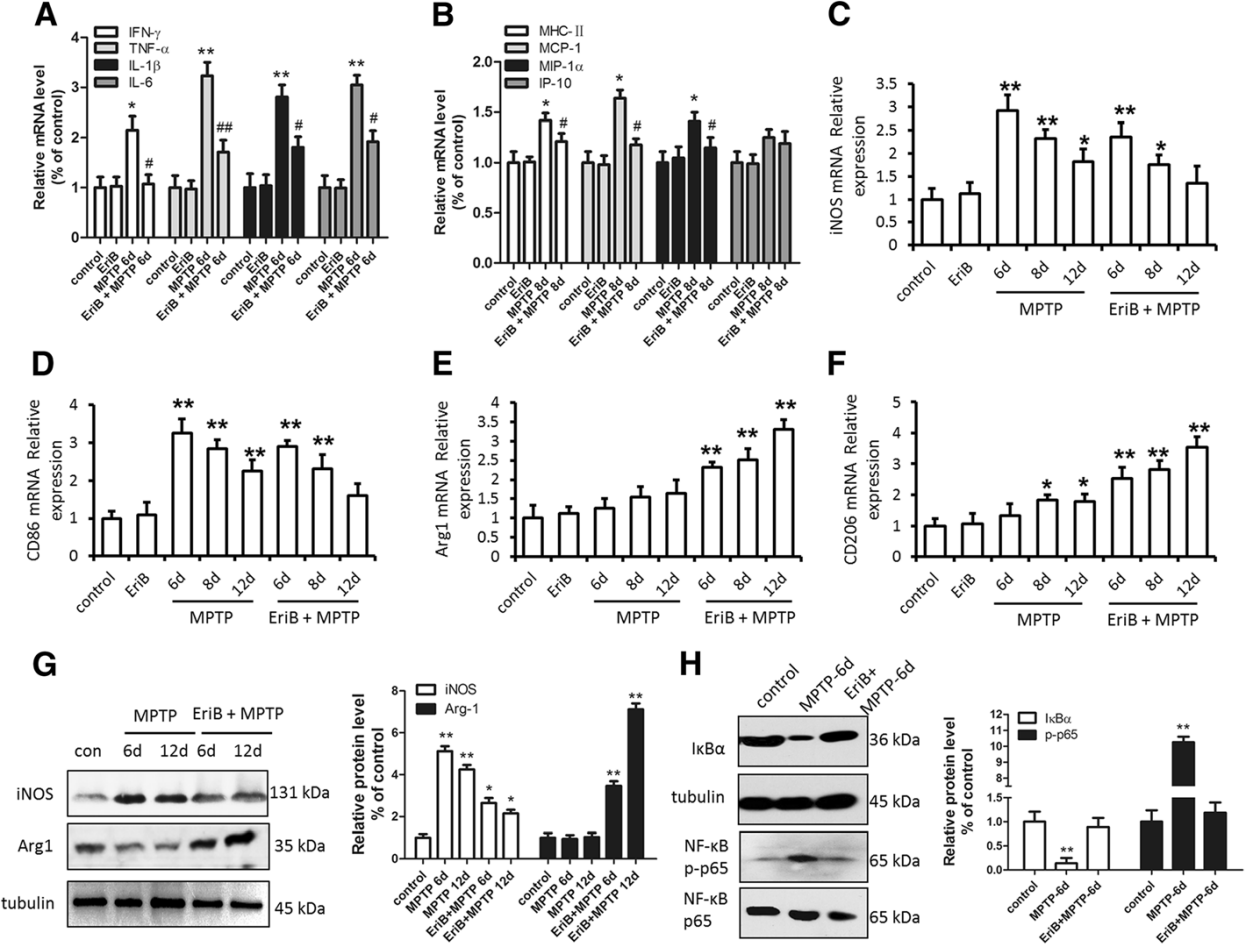
EriB reduced proinflammatory cytokines production and shifted microglia subtype in PD mice model. **a** mRNA level of IFN-γ, TNF-α, IL-1β and IL-6 in SN area after MPTP and EriB injection on 6th day. **b** mRNA level of MHC-II, MCP-1, MIP-1α and IP-10 in SN area after MPTP and EriB injection on 8th day. iNOS (**c**), CD86 (**d**), Arg1 (**e**) and CD206 (**f**) mRNA level in SN area after MPTP and EriB injection on 6th day, 8th day and 12th day. **g** Western-blot detected the iNOS and Arg1 protein level in SN area after MPTP and EriB injection on 6th day and 12th day. **h** Western-blot analysis the IкBα, p65 and p-p65 level in SN area after MPTP and EriB injection on 6th day. *: vs control, #: vs MPTP group. *&#: *P*<0.05, **&##: *P*<0.01

In order to determine whether microglia is activated, we examined the expression of inflammatory cytokines, inflammatory cell surface antigens and microglia subtype markers in midbrain tissue of PD mouse. In contrast to MPTP-treated mice, EriB administration remarkably attenuated the expression of proinflammatory cytokines (IFN-γ, TNF-α, IL-1β, IL-6) and inflammatory cell surface antigen (MHC-II, MCP-1 and MIP-1α) (Fig. 3a, b) in EriB and MPTP-treated mice. In order to further determine the cause of microglia activation, we checked the expression levels of M1 subtype markers (inducible nitric oxide synthase [iNOS] and CD86) and M2 subtype markers (CD206 and Arginine 1 [Arg1]). The results showed that the mRNA levels of M1 subtype markers iNOS and CD86 were both higher in MPTP-treated mice than in normal control, on 6th day and 8th day following EriB and MPTP administration, iNOS and CD86 mRNA levels were also higher than normal control, however, on 12th day following EriB and MPTP administration, iNOS and CD86 mRNA levels decreased. Accordingly, M2 subtype markers CD206 and Arg1 mRNA levels were both high in EriB and MPTP-treated mice. (Fig. 3c, d, e, f). The trend of protein level of iNOS and Arg1 was consistent with its mRNA level (Fig. 3g). These results indicated that MPTP induced production of M1 microglia, and with administration of EriB, M1 microglia shift to M2 microglia gradually. Taken together, these data indicated that EriB could attenuate the activation of microglia in the early stage of MPTP mouse model by inhibiting cytokines and surface antigen expression, and by shifting M1 microglia to M2 microglia. In order to confirm the mechanism of EriB’s inhibiting the activation of microglia, we detected the expression style of p65, phosphorylated-p65 and IκBα with or without EriB and MPTP treatment, Western-blot analysis showed that IκBα protein level decreased and p-p65 protein level increased with MPTP stimulation only, however, EriB inhibited the increase of p-p65 expression caused by the decrease of IκBα expression in PD model. These results indicated that decreasing the phosphorylation of p65 was one of the critical factors of inhibiting activated microglia by EriB (Fig. 3h).

### EriB protected DA neurons by inhibiting microglia activation

**Fig. 4:**
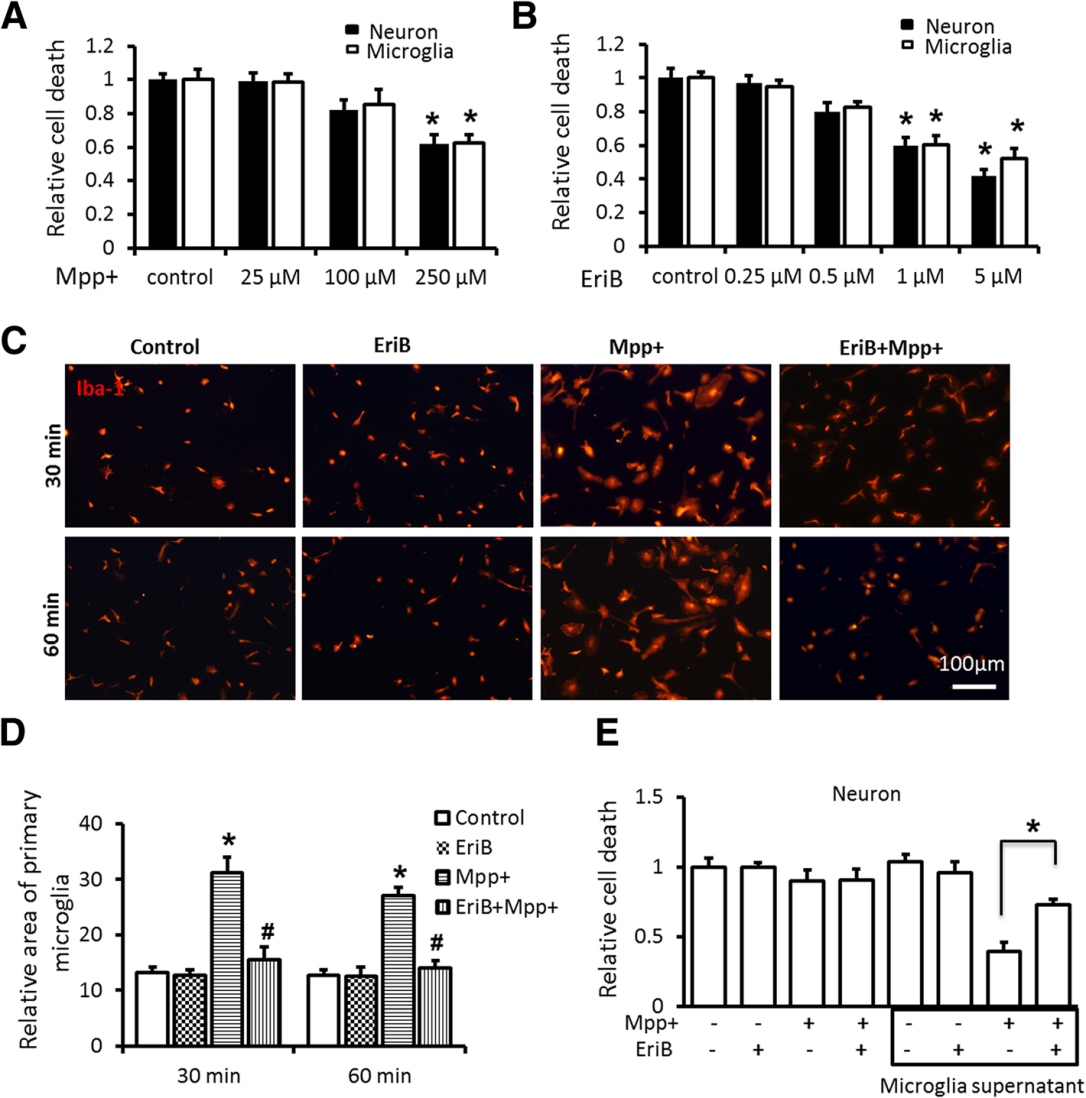
EriB protected DA neurons from MPP^+^ induced neurotoxicity by inhibiting microglia activation. **a** and **b** CCK-8 kit detected neuron and microglia cell death with different concentration of EriB and Mpp+. **c** Immunocytochemical staining for Iba-1 positive cultured microglia after Mpp + and EriB stimuli at 30 min and 60 min. **d** The relative size of microglia of immunocytochemical staining for Iba-1 positive cell after Mpp + and EriB stimuli. **e** CCK-8 assay detected neuron death after microglia supernatant stimuli. Scale bar: 100 μm. *: vs control, #: vs Mpp + group. *&#: *P*<0.05

**Fig. 5:**
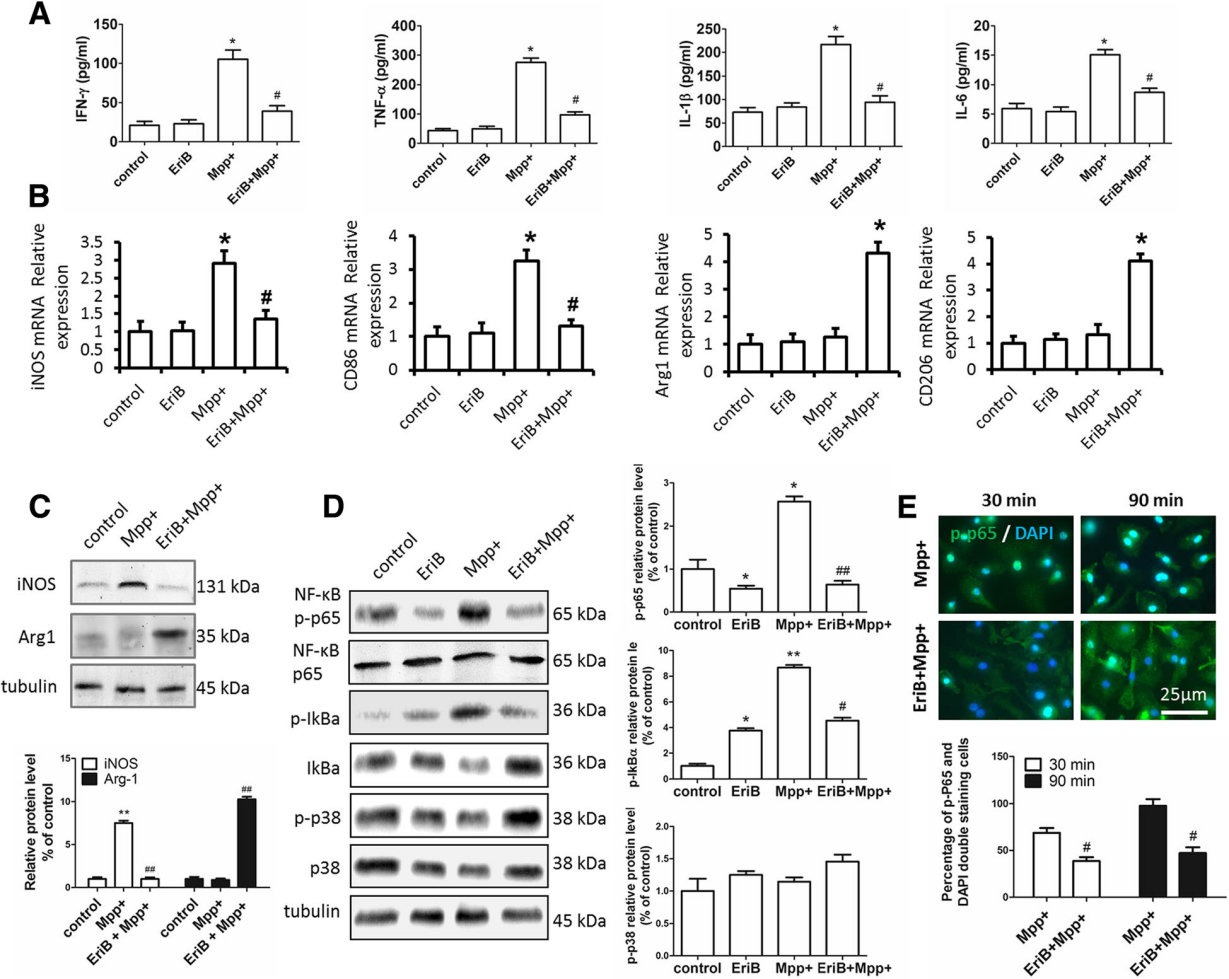
Effect of EriB on inhibiting microglia activation was mediated via the NF-κB signaling pathway. **a** protein level of IFN-γ, TNF-α, IL-1β and IL-6 after Mpp + and EriB stimuli. **b** iNOS, CD86, Arg1 and CD206 mRNA level after Mpp + and EriB stimuli. **c** Western-blot detected the iNOS and Arg1 protein level after Mpp + and EriB stimuli. **d** Western-blot analysis the NF-κB signaling pathway of cultured microglia after Mpp + and EriB stimuli. **e** immunocytochemical staining for p-p65 of cultured microglia after Mpp + and EriB stimuli, and statistical analysis of the percentage of p-p65 and DAPI double staining in cultured microglia after Mpp + and EriB stimuli. Scale bar: 25 μm. *: vs control, #: vs Mpp + group. *&#: *P*<0.05, **&##: *P*<0.01

To test whether DA neurons damage could be prevented by EriB-mediated inhibition of microglia activation and associated proinflammatory cytokines secretion, first of all, we examined the degree of neuron and microglia damages with different concentration of EriB and MPP^+^ separately by cell viability assay, the results revealed that the concentration of MPP^+^ greater than 250 μM could cause significant neuron and microglia damages (Fig. 4a), and the concentration of EriB greater than 1 μM could cause significant neuron and microglia damages similarly (Fig. 4b). Accordingly, 25 μM MPP^+^ and 0.25 μM EriB was respectively used to study the role of EriB on the inhibition of activated microglia and associated DA neurons protection. We firstly examined the inhibition role of EriB in primary cultured microglia, histological analysis showed the results that 0.25 μM EriB could keep microglia in resting status even if microglia was stimulated by 25 μM MPP^+^ (Fig. 4c, d). Then, we detected the death rate of neurons which was treated with or without microglia conditioned supernatant, in which microglia was in resting state or activated by MPP^+^. As shown in Fig. 4e, microglia stimulated by 25 μM MPP+ induced the 58% death rate of neurons, while EriB decreased the death rate to 34% of neurons. These results suggested that EriB might act as an inhibitor for the activation of microglia and the death of neurons might be induced by secreted inflammatory cytokines from activated microglia. Therefore, In the meantime, we detected the levels of inflammatory cytokines such as IFN-γ, TNF-α, IL-1β and IL-6. As shown in Fig. 5a, 0.25 μM EriB administration in cultured primary microglia greatly reduced the protein levels of IFN-γ, TNF-α, IL-1β and IL-6 induced by 25 μM MPP^+^, suggesting that EriB could inhibit the secretion of inflammatory cytokines of microglia to protect DA neurons. We also detected the subtype of microglia in cultured microglia, consistent with in vivo results, the expression levels of M1 subtype markers iNOS and CD86 were high and M2 subtype markers CD206 and Arg1 were low in MPP^+^ treated microglia, and iNOS and CD86 were low and CD206 and Arg1 were high in EriB treated microglia (Fig. 5b, c). These results confirmed that EriB protected neuron damage from shifting the subtype of microglia. To confirm whether EriB targeted inhibition of microglia activity was mediated via the NF-κB signaling pathway, we also detected the protein expressional level of p-p65 by Western-blot. As shown in Fig. 5d, EriB significantly reduced the activity of p-p65 and p-IκBα, however, EriB could not alter obviously the activity of p-p38.

### EriB inhibited p65 phosphorylation and p-p65 nuclear translocation

To further confirm whether EriB targeted inhibition of microglia activity was mediated via the NF-κB signaling pathway, we detected the protein expression and p-p65 location by western-blot and immunofluorescence in vitro. As shown in Fig. 5d, EriB significantly reduced the activity of p-p65 and p-IκBα, however, EriB could not alter obviously the activity of p-p38.The results of immunofluorescence indicated that EriB inhibited the generation and nuclear translocation of p-p65 induced by MPP^+^ (Fig. 5e). Taken together, our findings indicated that EriB-mediated regulation of microglial activation involved the DA neurons protection in PD mouse model, and NF-κB signaling pathway may influence inflammation-induced DA neurons damage.

## Discussion

PD is characterized by the loss of DA neurons in the SNpc neurons and often by the formation of aggregated α-synuclein which is the major component of the intracytoplasmic lewy body inclusion, which has been shown to promote neuroinflammation. The level of cytokines and chemokines was persistently elevated by chronically activated microglia, which may contribute to hastening neuronal dysfunction and degeneration of DA neurons [23, 24]. Therefore, how does the dysfunction of microglial lead to the pathogenesis of PD need to be researched. Previous studies observed microglia state in post mortem specimens only in the terminal hours of PD patients, however, whether microglia had been activated in early stage of PD remains completely unknown. Some studies reported that if microglia was activated in vitro, and what subtype of microglia in damaged tissue, it could contribute to the death of DA neurons [25, 26], but the specific mechanism remains to be studied. In MPTP mouse model, we found that with microglia activation, the mRNA levels of IFN-γ, TNF-α, IL-1β and IL-6 were increased at the 6th day post MPTP injection, activation associated proteins of microglia such as MHC-II, MCP-1 and MIP-1α were all up-regulated at the 8th day after MPTP injection. Inflammation-mediated neurotoxicity in PD can occur as a consequence of microglial subtype shift and over activation, the phosphorylation of NF-κB might be the signaling regulating molecular of microglia activation in the early stage. We focused on the study of DA neuronal damage induced by inflammatory cytokines released from microglia cells, in order to further define the role of microglia in the progression of PD, and also to investigate whether the inhibition of activated microglia and inflammatory cytokines was an important way to protect DA neurons.

Eriocalyxin B (EriB) is a diterpenoid extracted from *Isodoneriocalyx*, which has been used as an anti-inflammatory remedy and was shown to have a role in antitumor effects and anti-infection [11]. Lu *at al.* reported that EriB ameliorates inflammatory/autoimmune disorders by suppressing T helper (Th) 1 and Th17 cells, and the underlying molecular mechanism was involved in activator of NF-κB signaling pathways. Their study also demonstrated that EriB could block TNF-induced NF-κB activation by inhibiting IκB degradation in acute myeloid leukemia cell [16]. Previous study simultaneously indicated EriB could elevate ROS level by modulating NF-κB signaling pathways [12]. Our study uniquely shows that EriB could provide neuronal protection against the excessive activation of microglia in MPTP mouse model via NF-κB signaling pathways. EriB treatment not only attenuated the severity of neuron damage but also relieved motor deficits of MPTP mouse model. Indeed, EriB markedly decreased the expression of cytokines and inhibited the activation of microglia, which was the key point of reducing neuronal loss. Although mounting evidence indicates that microglial activation contributes to neuronal damage in PD, how to evaluate and define the activated and over-activated microglia need an in-depth research.

Although the majority of studies indicate that microglial activation was harmful for neurons, the inflammatory state of microglia was associated with not only neurotoxic consequences but also neuroprotective effects [27, 28]. In the MPTP mouse model, the neurotoxin stimulates a transient increase of macrophage infiltration and concomitant with microglia activation, suggesting that inflammatory reaction may play an important role in PD progression [29]. Lipopolysaccharide-induced cytokines and chemokines which produced by microglia in turn bind to microglial receptors could potentiate microglia activation [30]. A study showed that neutralizing antibodies to IL-1β or TNF-α could attenuate at least 50% DA neuronal death induced by LPS in primary cultures [31]. Taken together, these findings demonstrated that cytokines released from activated microglia play an important role in DA neuronal degeneration and PD pathogenesis. Microglia can exert beneficial or harmful effects, and this mainly depends on the two different microglia subtypes. Pro-inflammatory M1 microglia via classical pathways disrupts the internal environment, in contrast, anti-inflammatory M2 microglia via selective pathways plays a neuroprotective role. In the present study, IL-1β and TNF-α expression were detected not only in mouse SNpc but also in cultured microglia which were induced by MPP^+^, and in vitro study revealed cytokines released from activated microglia could directly lead to DA neurons damage or death except for MPP^+^ neurotoxicity. DA neurons damage or death could be prevented by EriB treatment due to blocking TNF-induced NF-κB activation. Therefore, with the evidence accumulation, the determined role of EriB on excessive neuro-inflammation in PD pathogenesis should gradually attract more and more attention. In the present study, we provide evidence that DA neurons loss and hypokinesis were associated with activation of microglia in PD mouse model, similar observations in cultured microglia suggested that activation of microglia by MPP^+^ enhanced the production of IFN-γ, TNF-α, IL-1β, IL-6 and induced the additional M1 microglia, at the same time, high level of main histocompatibility complex-II (MHC-II), monocyte chemotactic factor-1 (MCP-1) and macrophage inflammatory protein-1α (MIP-1α) were also detected. We also confirm that activated microglia rather than MPP^+^ led to a large number of DA neurons death. However, in the presence of EriB, DA neurons damage reduced because the harmful M1 microglia shifted to the neuroprotective M2 microglia with decreased pro-inflammatory cytokines which were secreted by M1 microglia. Furthermore, these effects were associated with phosphorylation of transcription and nuclear factor-κB (NF-κB), which entered the nucleus and activated the inflammatory cytokines signaling pathways. In summary, we concluded that EriB was able to exert DA neurons protection roles in PD model by targeting p65 phosphorylation of microglia. As the pathogenesis of PD often involves a complicated network of multiple factors, compounds from traditional Chinese medicine such as EriB, which exhibit multiple influences against inflammation, might need more attention, and might provide promising therapeutic approach for PD.

## Limitations of the study

This work is mostly confirmed that EriB exerts potent anti-inflammatory effects through selective modulation of microglia activation by targeting NF-κB signaling pathways, thus exerting the protective effect against on MPP + −induced DA neurons injury. However, we don’t directly confirm that activated microglia is a big risk factor to DA neurons in vivo although it is hard to be confirmed. Fortunately, we found that EriB indeed play a protective role to damaged DA neurons.

## Abbreviations

Arg: Arginine
CNS: Central nervous system
CSF: Cerebrospinal fluid
DA: Dopaminergic
DIV: Day in vitro
EriB: Eriocalyxin B
FBS: Fetal bovine serum
IFN: Interferon
IL-1β: Interleukin-1
iNOS: Inducible nitric oxide synthase
MPTP: 1-methyl − 4-phenyl-1,2,3,6-tetrahydropyridine
NF-κB: Nuclear factor-κB
PD: Parkinson’s disease
SN: Substantia nigra
SNpc: Substantia nigra pars compacta
TGF: Transforming growth factor
TH: Hydroxylase
TNF-α: Tumor necrosis factor-alpha
